# Salud pública y la interdependencia digital: evolución tecnológica, sostenibilidad tecnológica y la revolución del usuario

**DOI:** 10.26633/RPSP.2021.156

**Published:** 2021-12-16

**Authors:** Marcelo D’Agostino, Myrna Marti, Felipe Medina Mejia, Victoria Malek, Sebastián García Saiso

**Affiliations:** 1 Organización Panamericana de la Salud Organización Panamericana de la Salud Washington D.C Estados Unidos de América; 2 Consultora internacional Consultora internacional Argentina; 3 Consultor internacional Consultor internacional Colombia

**Keywords:** Acceso a la información, interoperabilidad de la información en salud, acceso a las tecnologías de la información y comunicación, Access to information, health information interoperability, access to essential medicines and health technologies, Acesso à informação, interoperabilidade da informação em saúde, acesso a medicamentos essenciais e tecnologias em saúde

## Abstract

Se plantea que la llamada “revolución tecnológica en el sector salud”, debido al auge del uso de las tecnologías de la información y comunicaciones (TIC) que ocurrió durante la pandemia de COVID-19, se debe, en verdad, a una revolución del usuario que, por su estrecha relación con las TIC y en el punto de inflexión de la pandemia, ha movilizado a los sistemas de salud. Al respecto, se plantea un modelo conceptual de evolución y revolución tecnológica de los usuarios, con transiciones de la aceptación de la salud digital al entendimiento de su potencial, así como de la sostenibilidad de la salud digital a la confianza en sus diversas aplicaciones y gobernanzas. Lo anterior requiere de enfoques y acuerdos claros entre los diferentes sectores del sistema de salud en materia de gerencia, infraestructura, políticas y capacitación, entre otros, centrados en la revolución del usuario y asegurando no dejar a nadie atrás. El presente artículo pretende conceptualizar el proceso de evolución y revolución de las TIC aplicadas a la salud en el contexto de la pandemia de COVID-19.

## Abstract (en)

The "technological revolution in the health sector" resulting from the boom in the use of information and communications technologies (ICT) during the COVID-19 pandemic may, in fact, be due to a revolution among users whose close relationship with ICT has mobilized health systems, with the pandemic as a turning point. This article proposes a conceptual model of technological evolution and revolution among users, with transitions from acceptance of digital health to an understanding of its potential, and from the sustainability of digital health to trust in its various applications and governance. This requires clear approaches and agreements between the different sectors of the health system in terms of management, infrastructure, policies, and training, among other areas, focused on the user revolution and ensuring that no one is left behind.

This article aims to conceptualize the process of evolution and revolution in ICT, as it applies to health in the context of the COVID-19 pandemic.

## RESUMO

Argumenta-se que a chamada “revolução tecnológica no setor da saúde”, devido ao boom no uso das tecnologias da informação e comunicação (TIC) ocorrido durante a pandemia de COVID-19, na verdade se deve a uma revolução do usuário que, devido à sua estreita relação com as TIC e no ponto de inflexão da pandemia, mobilizou os sistemas de saúde. Nesse sentido, propõe-se um modelo conceitual de evolução e revolução tecnológica dos usuários, com transições da aceitação da saúde digital para a compreensão do seu potencial, bem como da sustentabilidade da saúde digital para a confiança em suas diversas aplicações e governanças. Isso requer enfoques e acordos claros entre os diferentes setores do sistema de saúde em termos de gestão, infraestrutura, políticas e capacitação, entre outros, com foco na revolução do usuário e assegurando que ninguém fique para trás. Este artigo tem como objetivo conceitualizar o processo de evolução e revolução das TIC aplicadas à saúde no contexto da pandemia de COVID-19.

Existe un sesgo de percepción que llama “revolución tecnológica en el sector salud” al auge del uso de las tecnologías de la información y comunicaciones (TIC, término amplio en el que convergen las tecnologías empleadas para la información, las telecomunicaciones, la computación, la transmisión, el almacenamiento y la compartición, entre otros) como consecuencia de la pandemia de COVID-19. Sin embargo, se trata en gran medida de una revolución del usuario quien, por su estrecha relación con las TIC, ha movilizado a los sistemas de salud. Si bien la pandemia ha acelerado aún más la aplicación de las TIC en salud y en salud pública, es imperativo desarrollar mecanismos de gobernanza para avanzar de manera sostenida y sostenible, de manera que puedan capitalizarse todas estas innovaciones en el contexto actual de forma equitativa e inclusiva para la mejora de la salud de las personas.

El uso de las TIC en salud para dar respuesta a emergencias y servicios en salud existe desde antes de la pandemia de COVID-19 (1). En el cuadro 1 se mencionan algunos ejemplos sobre cómo algunas tecnologías, que ahora se consideran emergentes en el área de la salud, ya se aplicaban antes (2-9).

Por otro lado, previo a la pandemia, ya existía un contexto tecnológico fértil con potencial para ser capitalizado en diversos sectores de la sociedad, en particular la salud pública. Por ejemplo, en el 2010, ya el mundo contaba con más de 76 suscripciones a teléfonos celulares por cada 100 personas (hoy en día, esa cifra es de 104 por cada 100 personas), casi 29% de la población hacía uso de internet (el porcentaje actual es de 46%), y 517 millones de personas eran usuarias de Facebook^®^ (hoy son más de 2260 millones) y 480 millones de YouTube^®^ (hoy son aproximadamente 1900 millones) (10).

**CUADRO 1.** Primeras aplicaciones en salud de tecnologías consideradas emergentes

Tecnologías de información y la comunicación	Año de inicio	Descripción	Ejemplo de uso en la pandemia
Inteligencia artificial	1950	Uso de computadoras para simular el comportamiento inteligente y el pensamiento crítico	Realidad aumentada por inteligencia artifical en la radiología de tórax para distinguir COVID-19 de la neumonía de otro origen Simulación, modelos y predicción de brotes.
Chatots	1966	Primer chatbot desarrollado por el Instituto Tecnológico de Massachusetts ELIZA	Triaje de síntomas y orientación en salud Solicitud de turnos
Robótica	1966	Shakey, la primera “persona electrónica”, fue desarrollada en el Instituto de Investigación de Stanford	Desinfección de superficies, medición de temperatura en áreas públicas
Telesalud	1988	Filmación de cirugía y posterior retransmisión en Argentina	Teleconsultas y teleeducación
Cadena de bloques (*blockchain*)	1991	Cadena de bloques criptográficamente segura	Gestión de registros médicos electrónicos
mSalud	2006	Prestación de asociados a la salud mediante dispositivos móviles	Asistencia telefónica para la asistencia sanitaria Servicios telefónicos de emergencia gratuitos

COVID-19: enfermedad por el coronavirus del 2019 (por su sigla en inglés).

***Fuente:*** elaboración propia.

Ha existido una creciente población usuaria de TIC, con necesidades de salud evidenciadas y documentadas. En este sentido, los usuarios (pacientes, trabajadores de la salud, cuidadores y la población general) han utilizado las TIC para gestionar y enfrentar situaciones en salud particulares o comunitarias de diferentes formas: a) desarrollo y consumo de productos informativos disponibles en internet y redes sociales; b) creación de comunidades virtuales; c) consultas por teléfono, correo electrónico y mensajes instantáneos a trabajadores de la salud; d) uso de verificadores de síntomas en internet para mejorar la toma de decisiones; y e) verificación de redes de servicios de salud en internet, entre otros muchos ejemplos.

El presente artículo pretende conceptualizar el proceso de evolución y revolución de las TIC aplicadas a la salud en el contexto de la pandemia de COVID-19.

## MODELO CONCEPTUAL

**FIGURA 1.**
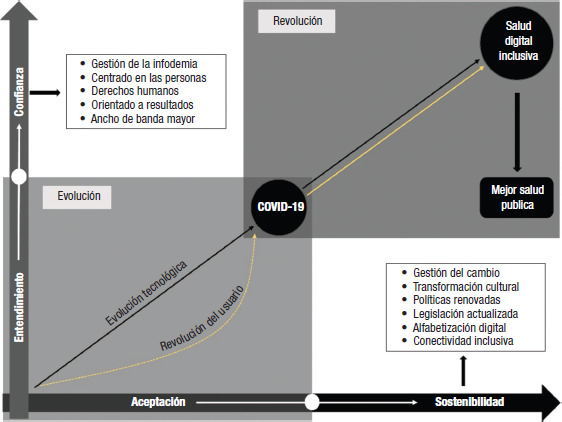
Modelo conceptual de la evolución y revolución de las tecnologías aplicadas a la información y la comunicación aplicadas a la salud en el contexto de la pandemia de COVID-19

En la figura 1 se aprecia cómo la pandemia de covid-19 ha sido un artífice clave en el paso de una evolución de las tecnologías, y de su uso por parte de los usuarios o revolución del usuario, a una revolución completa. Para que este cambio tenga lugar, a medida que se logra la aceptación inicial, gracias a los impactos positivos que muestra la salud digital, aumenta el entendimiento de sus aplicaciones. En esa misma relación, a medida que se hace más sostenible, la salud digital se integra en el sistema de salud y en la sociedad; de esta manera, aumenta la confianza de los usuarios sobre estos servicios y productos. Todo esto ocurre mediante la adecuada convergencia de actividades y enfoques centrales como, por ejemplo, la gestión de la infodemia y del cambio.

### Evolución

El avance continuo en las TIC, así como el creciente acceso informado de la población a ellas, ha creado un entorno fértil de innovación que permite a los usuarios desarrollar de forma independiente soluciones digitales para la gestión de algunas condiciones de salud. Este conjunto de factores causa una revolución y genera un círculo virtuoso de desarrollo tecnológico pensado y centrado en las personas.

Esta revolución pone al usuario en el centro de la escena y se da en un contexto donde, cuanto mayor ha sido la aceptación, en algunos casos por interés y en otros por falta de opciones, mayor ha sido el entendimiento sobre el potencial beneficio que trae el mundo digital a la salud pública.

Sin embargo, es necesario apoyar esta revolución con medidas para transitar desde el período de aceptación a un período de sostenibilidad, así como del momento del entendimiento a un momento de plena confianza por parte de todos los actores involucrados.

### Revolución

Estamos transitando una nueva revolución: la revolución de los usuarios, miembros de una sociedad digital interconectada conocida como la Cuarta Revolución Industrial (un mundo donde las personas se mueven entre los dominios digitales y la realidad fuera de línea con el uso de tecnología conectada para habilitar y administrar sus vidas) (11), en la que existen procesos de trabajo en red para la cocreación de bienes públicos digitales y se eliminan fronteras y barreras de acceso a los posibles beneficios de las TIC en la salud de las personas.

Sin embargo, para que una revolución sea efectiva, debe tenerse una comprensión adecuada del presente y del pasado, ya que la velocidad de los avances no siempre va de la mano con la capacidad de una asimilación masiva, principalmente en el sector salud. Por ello, se hace necesario establecer las bases fundacionales que aseguren su sostenibilidad y su efecto positivo. Asimismo, es necesario que las TIC en salud estén siempre ponderadas en función de las otras metas en salud, de forma que se conviertan en una herramienta para alcanzar esas metas y no en un objetivo en sí mismo.

## LAS TRANSICIONES

### Aceptación.

Durante la pandemia de COVID-19, el aislamiento preventivo de las personas, sumado al cierre de fronteras y de casi todos los servicios no indispensables, fue una de las intervenciones ejecutadas con el objetivo de reducir del ritmo de contagio. Esto ha llevado a una apropiación acelerada de las TIC como uno de los medios principales de interacción con la sociedad. Es importante anotar que la adopción de una u otra TIC depende, en gran medida, del nivel de acceso y de la capacidad de uso. Es decir, no todas las TIC se han utilizado del mismo modo y con la misma intensidad, para enfrentar los impactos sociales, económicos y de salud pública de la pandemia, ni de forma homogénea en los diferentes sectores poblacionales y distintos países.

***Fuente*:** elaboración propia.

### Entendimiento.

A medida que ha aumentado la aceptación de las TIC en salud, y de la salud digital como un todo, también ha aumentado el entendimiento sobre el potencial de las TIC, y con ello, mayores serán los beneficios de su uso para la salud y la salud pública.

### Sostenibilidad.

La incorporación sostenible de TIC en la toma de decisiones del sector salud, así como en la formación temprana y continua de recursos humanos, permite profundizar en los procesos democratizadores, de aseguramiento de la calidad y consideraciones éticas de las políticas públicas (12). A su vez, para lograr la sostenibilidad se necesitará de una sistematización y reingeniería de procesos basada en la experiencia práctica y estudios empíricos de los resultados observados sobre la aplicación de las TIC en los diferentes ámbitos.

### Confianza.

La sostenibilidad es clave para promover la confianza entre los actores del sistema de salud y de la sociedad como un todo. Por ejemplo, en el acto médico, es indispensable la confianza mutua, no solo entre el paciente y el profesional tratante, sino confianza en la robustez del sistema de salud, la calidad de las prestaciones y en el medio -ahora digital- donde se realizan.

## LOS ENFOQUES

### Gestión del cambio.

Las competencias básicas para la salud pública en la era de la interdependencia digital requieren enfoques renovados, sobre todo en los aspectos relacionados con la adopción de las TIC. Sin embargo, los tomadores de decisiones del sector salud aún no incorporan estos temas como parte de una cultura de trabajo, y esto solo podría lograrse con una estrategia formal de gestión del cambio. La gestión del proceso de cambio dentro de las organizaciones de salud pública es importante, porque la gestión adecuada y sistemática del cambio está vinculada a un mejor desempeño organizacional (13).

### Transformación cultural.

Es necesario construir actores sociales con una participación activa que vaya desde el individuo aislado al sujeto que se reconoce como parte de una comunidad. Este sujeto, como construcción colectiva, deberá estar integrado principalmente por los trabajadores de la salud, los profesionales y los usuarios; es decir, por la sociedad en general, dado que todos somos potenciales usuarios de salud. En este sentido, las TIC deben ser utilizadas como herramientas habilitadoras del cambio en donde todos los actores pasan a ser partícipes activos y socios en los procesos.

### Políticas renovadas.

La apropiación de las TIC, que implica un acercamiento y un uso con propósito e informado, debería ser un componente transversal de las políticas públicas del sector salud. Sin embargo, no todas las políticas o planes de salud actuales le dan el lugar necesario a las TIC y, por lo tanto, se hace necesario acelerar un proceso de revisión y modernización de estas, con la inclusión de agendas o estrategias digitales nacionales. Además, se deben considerar los enfoques multisectoriales y multidisciplinarios desde la construcción hasta su aplicación.

### Legislación actualizada.

La revolución del usuario y la salud digital constituyen grandes desafíos para los instrumentos normativos existentes. Se deben considerar las regulaciones relativas a la protección de datos personales y su aplicación en salud, vinculado a la privacidad, confidencialidad y privacidad de los datos. Esto es fundamental en temas como la adopción de plataformas de teleconsulta y el análisis de datos en salud, para su certificaicón mediante estándares de seguridad y confiabilidad. Esto debe ser respaldado por instrumentos normativos adecuados y acordes a una sociedad digitalmente interconectada (14).

### Alfabetización digital.

Es un proceso constante y permanente de aprendizaje que, hoy en día, está limitada al uso de las TIC y las diferentes competencias requeridas para la generación y evaluación de contenido diverso. En este sentido, es fundamental comprender y fomentar diferentes habilidades conforme a las necesidades de cada sector: el paciente, que también es usuario (aprender cómo se usan), el tomador de decisión (saber para qué se usan) o el desarrollador y generador de contenido (comprender cómo llegar a más usuarios).

### Conectividad inclusiva.

Las TIC tienen el potencial de reducir las desigualdades en salud al permitir que las personas accedan a información y herramientas digitales de prevención y cuidado en el momento y el formato adecuado. La inclusión digital implica el acceso apropiado y la adquisición de habilidades digitales, así como consideraciones de usabilidad y navegabilidad (UX/UI, por su sigla en inglés) en el desarrollo de soluciones tecnológicas.

### Gestión de la infodemia.

El acceso a información confiable en el momento y el formato requerido, que responda a necesidades específicas y culturalmente pertinentes, representa uno de los mayores desafíos para la generación de confianza en las políticas públicas y decisiones relacionadas con la salud, así como para la adopción segura de TIC en procesos de autocuidado o de continuidad de tratamientos a distancia (15).

### Salud digital centrada en las personas.

Es esencial diseñar una hoja de ruta que oriente las acciones para que la revolución del usuario cree una salud digital centrada en las personas. Es necesario reducir algunas barreras que se han hecho evidentes en la pandemia de COVID-19, como la oferta desigual de teleconsultas y otros servicios de salud digital que no han respondido de forma uniforme ni han considerado las particularidades de contextos sociales específicos.

### Derechos humanos y derechos en salud.

En la era de la interdependencia digital, el enfoque se derechos humanos requiere acciones inmediatas y profundas, como la revisión y la modernización de instrumentos jurídicos y normativos que tienen relación directa o indirecta con el sector salud. Las actividades para ejecutar iniciativas deben respetar siempre los principios de equidad, de derechos humanos, y de responsabilidad y sostenibilidad, para garantizar que tanto las personas como las comunidades accedan a servicios de salud de calidad sin sufrir dificultades financieras (16).

### Salud digital orientada a los resultados.

El modelo de atención centrado en los resultados propone orientar a los integrantes de los equipos de salud en la creación conjunta de planes de atención centrados en las personas que respondan a las prioridades, las necesidades, las preferencias y los valores compartidos (17). Este modelo debe integrarse a un modelo de salud digital también orientada a resultados, de manera que exista una retroalimentación continua entre ambas estructuras.

### Ancho de banda.

Las tecnologías anteriores a 5G presentan un marco poco satisfactorio para alcanzar los resultados establecidos en las políticas públicas de salud con el enfoque de no dejar a nadie atrás. La tecnología de comunicación 5G no solo puede lograr una transmisión de alta calidad de datos asociados a la salud como imágenes en 3D, sino que permite mejorar el diagnóstico y tratamiento remotos, entre otras aplicaciones (18).

## CONCLUSIONES

Los sistemas de salud resilientes deben incorporar a la salud digital contextualizada en la revolución del usuario, con el fin de dar respuesta a las necesidades y los objetivos en salud, en particular en la salud pública. Las TIC tienen un papel estratégico en los esfuerzos individuales, comunitarios, locales, regionales y mundiales de salud en general y salud pública en particular para mejorar los sistemas de salud y para minimizar los efectos económicos y sociales de emergencias como la pandemia de COVID-19. Es prioritario que las poblaciones tengan acceso a redes y servicios digitales de salud; sin embargo, no dejar a nadie atrás en la era digital requiere llegar, además de a las poblaciones en situación de mayor vulnerabilidad social, económica, geográfica o cultural, también a aquellos que no tienen alfabetización digital. Se recomienda orientar el posicionamiento sostenible del sector salud en la era de la interdependencia digital hacia el desarrollo y aplicación de políticas públicas renovadas que logren la comprensión y consideración total de todas las características que trae esta nueva forma de sociedad global. Dichas políticas deberán ser sustentadas por instrumentos normativos que contengan, como mínimo, un marco de aplicación de banda ancha que describa cómo todos los habitantes podrán conectarse a internet en el futuro.

## Declaración.

Las opiniones expresadas en este manuscrito son responsabilidad del autor y no reflejan necesariamente los criterios ni la política de la *RPSP/PAJPH* y/o de la OPS.
